# Depth-Dependent Variability in Ultrasound Attenuation Imaging for Hepatic Steatosis: A Pilot Study of ATI and HRI in Healthy Volunteers

**DOI:** 10.3390/jimaging11070229

**Published:** 2025-07-09

**Authors:** Alexander Martin, Oliver Hurni, Catherine Paverd, Olivia Hänni, Lisa Ruby, Thomas Frauenfelder, Florian A. Huber

**Affiliations:** 1 Institute of Diagnostic and Interventional Radiology, University Hospital Zurich, 8091 Zurich, Switzerland; 2 Institute of Anesthesiology and Perioperative Medicine, University Hospital Zurich, 8091 Zurich, Switzerland

**Keywords:** ultrasound attenuation imaging, steatosis, MASLD, hepatorenal index, liver fat quantification, ultrasound

## Abstract

Ultrasound attenuation imaging (ATI) is a non-invasive method for quantifying hepatic steatosis, offering advantages over the hepatorenal index (HRI). However, its reliability can be influenced by factors such as measurement depth, ROI size, and subcutaneous fat. This paper examines the impact of these confounders on ATI measurements and discusses diagnostic considerations. In this study, 33 healthy adults underwent liver ultrasound with ATI and HRI protocols. ATI measurements were taken at depths of 2–5 cm below the liver capsule using small and large ROIs. Two operators performed the measurements, and inter-operator variability was assessed. Subcutaneous fat thickness was measured to evaluate its influence on attenuation values. The ATI measurements showed a consistent decrease in attenuation coefficient values with increasing depth, approximately 0.05 dB/cm/MHz. Larger ROI sizes increased measurement variability due to greater anatomical heterogeneity. HRI values correlated weakly with ATI and were influenced by operator technique and subcutaneous fat, the latter accounting for roughly 2.5% of variability. ATI provides a quantitative assessment of hepatic steatosis compared to HRI, although its accuracy can vary depending on the depth and ROI selection. Standardised imaging protocols and AI tools may improve reproducibility and clinical utility, supporting advancements in ultrasound-based liver diagnostics for better patient care.

## 1. Introduction

One key application of ultrasound attenuation imaging is in the assessment of liver health, particularly in diagnosing metabolic dysfunction-associated steatotic liver disease (MASLD), previously known as non-alcoholic fatty liver disease (NAFLD) [1,2]. The prevalence of hepatic steatosis in Western populations is estimated to affect 20–30% of individuals and is associated with severe comorbidities [3], such as metabolic syndrome and cardiovascular disease [3,4,5]. Early detection and intervention are vital for preventing progression to more severe disease stages and improving long-term patient outcomes.

While liver biopsy remains the gold standard for diagnosing hepatic steatosis, it is invasive, expensive, and carries risks such as sampling error and procedural complications [6]. This only emphasises the necessity for non-invasive alternatives such as MRI and ultrasound-based techniques. MRI-based proton density fat fraction (MRI-PDFF) has proven to be an accurate, non-invasive method for assessing liver fat [7,8]; however, its high cost, limited availability, and lengthy scanning times can limit its use in routine clinical practice. Whereas ultrasound-based attenuation imaging offers significant advantages due to its practicality, cost-effectiveness, and ability to integrate into existing imaging workflows with real-time feedback. Ultrasound systems can now use attenuation imaging (ATI) to provide quantitative data on liver fat content through attenuation coefficients [9,10,11,12], allowing precise and reproducible measurements across patient groups.

The hepatorenal index (HRI), a commonly used method to evaluate hepatic steatosis [13,14], compares the echogenicity of the liver to that of the kidney on ultrasound images. Although widely used, HRI is prone to limitations, including susceptibility to operator-dependent variability and the potential for false-positive or false-negative results. Its reliance on visual assessment may lead to inconsistencies when categorising steatosis. Despite these challenges, HRI remains a clinical standard in many institutions due to its simplicity and widespread availability. However, comparisons with ATI in recent studies suggest ATI may offer improved sensitivity and specificity for characterising liver steatosis by directly quantifying the attenuation coefficient—a metric strongly correlated with liver fat content [8,11,14,15,16,17,18]. The present study leverages these attributes of ATI to evaluate its diagnostic potential in comparison to HRI, highlighting the strengths and limitations of each approach. Attenuation imaging is increasingly used as an ultrasound diagnostic technique for assessing liver tissue and disease processes, in particular, steatosis. Attenuation imaging quantifies the reduction in ultrasound signal strength, providing essential information about anatomical properties and pathological changes [19,20,21]. While attenuation imaging can be performed using magnetic resonance imaging (MRI) and ultrasound, recent advancements in ultrasound technology have made it a widely used modality due to its affordability, portability, and non-invasive nature.

This work adds to the body of literature assessing quantitative ultrasound imaging by investigating the reliability of ultrasound ATI and HRI. We examine confounders such as depth, percentage body fat, and Region of Interest size, as well as inter-operator variability, in a healthy volunteer cohort.

## 2. Methods

Approval for this prospective single-institution study was obtained from both the institutional review board and the local ethics committee (Kantonale Ethikkommission Zürich; KEK-ZH Nr. 2015-0323). All volunteers provided written informed consent before participation, and data collection with consent for participation, scientific analysis, and publication was obtained from each participant. All images were acquired by 2 experienced radiologists using the protocol below.

Thirty-three participants (19 female, 14 male) were recruited through local advertisements. The inclusion criteria for the study required adult healthy volunteers aged 18 to 65 with no history of liver or biliary disease, diabetes, or recent medication use that could affect liver function. Additionally, participants needed to have a BMI between 17 and 30 kg/m^2^, no history of liver-related surgery, trauma, or interventional procedures. Sex, age, BMI, and alcohol intake were assessed for all participants. Volunteers were required to fast for at least three hours before measurements.

Table 1 provides the characteristics of the 33 volunteers.

All ultrasound measurements were performed using a TUS-AI800 ultrasound scanner (Aplio i800, Canon Medical System Corporation, Otawara, Japan) equipped with the i8CX1 convex array (centre frequency 4 MHz).

During the examination, volunteers lay supine with their right arm positioned behind their head. Towels were used to drape the volunteers during scanning. Commercially available ultrasound gel (UL-01, Skintact, Healthcare, London, UK) was applied to the convex probe, which was placed in the intercostal window along the midclavicular or anterior axillary line.

### 2.1. Measurement Protocol

**Figure 1:**
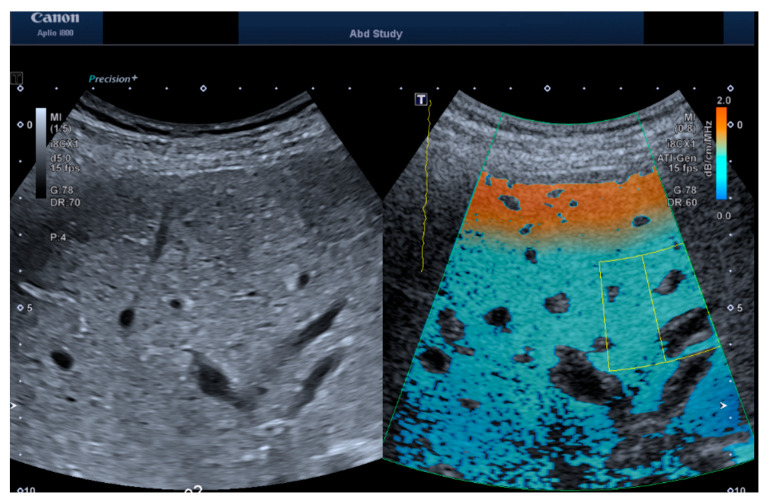
Exemplary images of b-mode and ATI measurements for both the convex probe. The ATI image shows the acquisition box placed (green box) and the Region of Interest (yellow box) placed at 3 cm depth.

The ATI mode was selected with a dual screen and a depth of 10 cm. The area of interest (AOI) box was enlarged to its maximum size and placed in the middle of the frame to encompass the subcutaneous fat, muscle, and liver. The volunteers were asked to inhale and slowly exhale, and an image was obtained and frozen in segment VI/VII. A region of interest (ROI) was first placed in an area 2 cm below the liver capsule with an R^2^ > 0.9 (Figure 1) to ensure the best image quality for further analysis at depth. Four additional repeated measurements were obtained by removing the probe and placing it in a similar area. A total of five repeat images were collected for examination. The volunteers were measured by an attending radiologist with 6 years of experience.

### 2.2. Depth and ROI Size Analysis

Two trained independent operators analysed the saved volunteer images offline after the ultrasound measurements. An assistant doctor with 4 years of experience and a postdoctoral researcher with 7 years of ultrasound experience analysed the images by placing regions of interest on them and changing the sizing of the areas. An attending radiologist with 15 years of ultrasound experience completed the final revision of the images.

For each image, two regions of interest were drawn at each depth below the liver capsule where possible. The depths were 2 cm, 3 cm, 4 cm and 5 cm. The ROI was sized at 2 cm in height and as narrow as possible, representing the “small ROI”, and at 2 cm in height and as wide as possible, where only liver tissue was present in the region of interest, representing the “large ROI”.

For each image, each volunteer’s surface of skin to liver capsule was also measured to quantify the subcutaneous fat to test as a confounding factor.

Following the data collection, each operator’s results were collated and compared for inter-operator comparison.

### 2.3. HRI Versus Attenuation Coefficient

**Figure 2:**
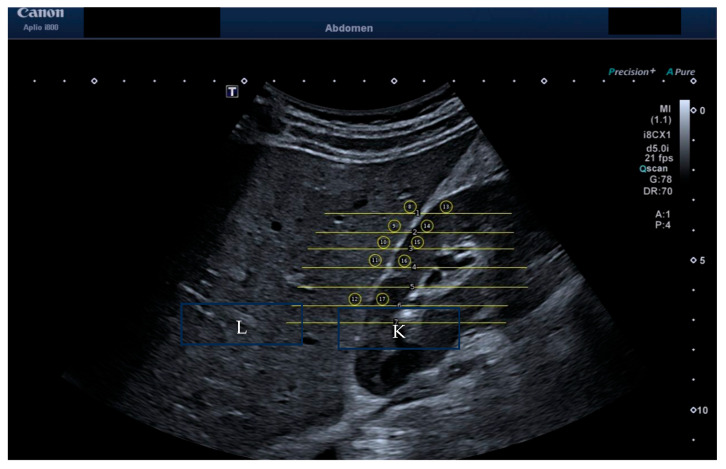
HRI measurement of brightness of liver versus kidney. The liver is labelled “L” and the kidney labelled “K” within the image.

For a complete analysis, as seen in Figure 2, the HRI for each volunteer was also measured by two independent operators using ImageJ software, version 1.54p (23): an assistant doctor with 3 years of experience and a postdoctoral researcher with 10 years of experience in ultrasound. The results were compiled, and an independent researcher analysed the measurement results.

### 2.4. Statistics

Statistical analysis was performed using GraphPad Prism 10.2.3. Unpaired *t*-tests were conducted to compare the measurement values for each volunteer across different methods or timeframes. Linear regression was used to assess the relationships between different measurement types. A *p*-value of less than 0.05 was considered statistically significant.

Intraclass Correlation Coefficient (ICC) Calculation to assess inter-operator reliability for HRI measurements. The ICC was calculated using a two-way random-effects model for absolute agreement, denoted as ICC(2,1). This model was selected as it accounts for both random operator effects and subject variability, making it appropriate for generalising results beyond the two raters included in this study. The ICC was computed using individual HRI measurements from both operators across all participants. The resulting ICC value was reported alongside the 95% confidence interval, and interpreted according to standard benchmarks: poor (<0.5), moderate (0.5–0.75), good (0.75–0.9), and excellent (>0.9) reliability. All statistical analyses were performed using GraphPad Prism 10.2.3.

## 3. Results

**Figure 3:**
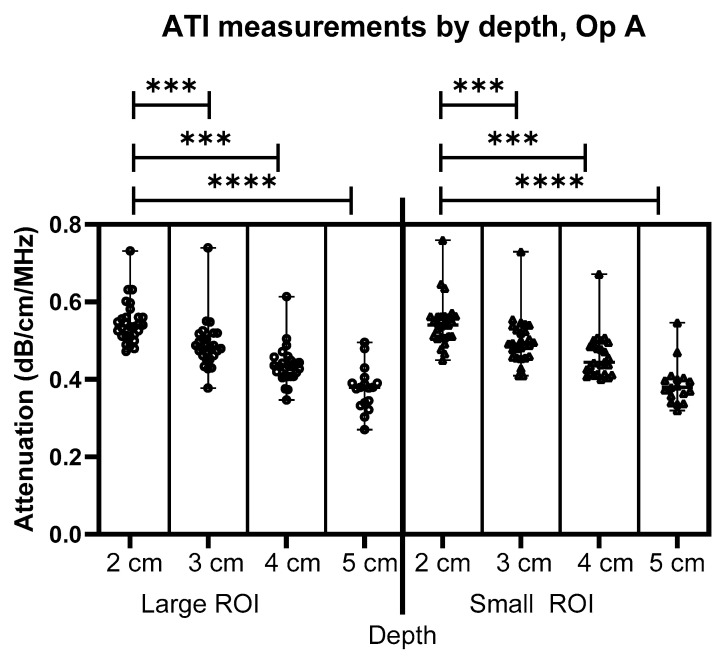
Comparison of Operator A image measurements of attenuation at 2–5 cm below the liver capsule for both large and small ROIs. *** = *p* ≤ 0.001 **** = *p* ≤ 0.0001.

**Figure 4:**
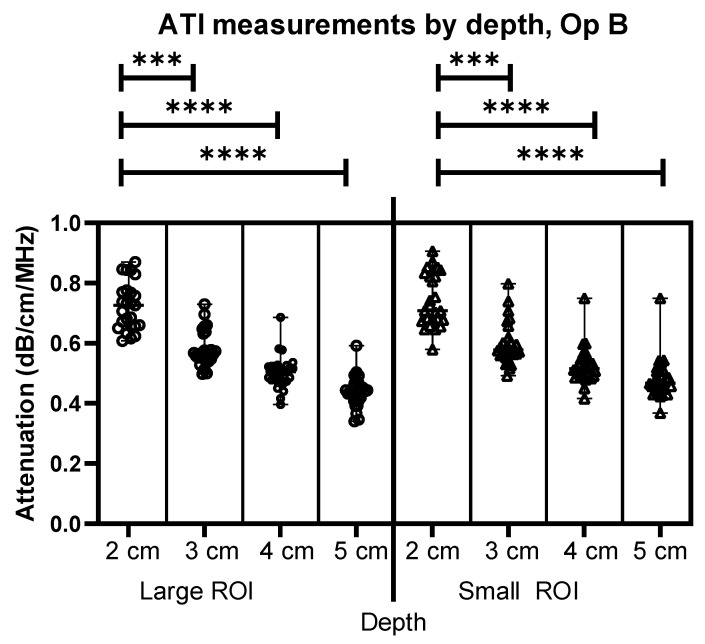
Comparison of Operator B attenuation measurements at 2–5 cm below the liver capsule for both large and small ROIs. *** = *p* ≤ 0.001 **** = *p* ≤ 0.0001.

**Figure 5:**
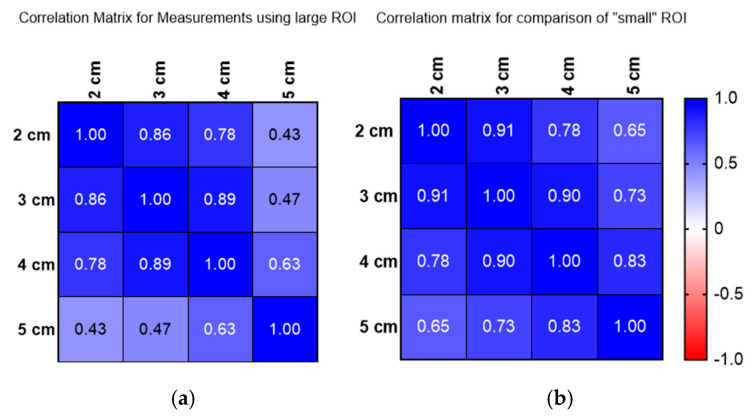
(**a**) Correlation matrix for large ROI measurements conducted 2–5 cm below the liver capsule. (**b**) Correlation matrix for small ROI measurements conducted 2–5 cm below the liver capsule.

Results for measurements from Operator A are shown in Figure 3, Figure 4 and Figure 5. Figure 3 compares the depths and size of interest for Operator A’s image measurements. As can be seen, there is a significant difference in depth alone for both the large ROIs and the Small ROIs, demonstrating a clear pattern and link between the depth of measurement and the achievable attenuation coefficient value. As measurement depth is increased, the attenuation coefficient value decreases at a rate of 0.05 dB/cm/MHz within the same liver (calculated using a simple linear regression model).

Figure 4 shows similar results to Figure 3, this time for Operator B’s images. The comparison between depths using different sizes of ROIs demonstrates an effect of depth for both Operators A and B. As can be seen, there is a significant difference in depth alone for both the large ROIs and the Small ROIs, demonstrating a clear link between the depth of measurement and the attenuation coefficient value, as the depth is increased, the attenuation coefficient value decreases at a rate of 0.05 dB/cm/MHz within the same liver.

Figure 5a,b show the correlation for measurements using a large ROI (a) and small ROI (b) and how these compare at depths of 2 to 5 cm. Figure 5 reveals that attenuation coefficient measurements significantly vary with depth, as demonstrated by both Operators A and B. Operator A’s measurements indicate a consistent decrease in attenuation values as depth increases, with a rate of approximately 0.05 dB/cm/MHz (0.054 dB/cm/MHz) within the same liver. This pattern was mirrored by Operator B, confirming a depth-dependent relationship across both large and small regions of interest (ROIs)

**Figure 6:**
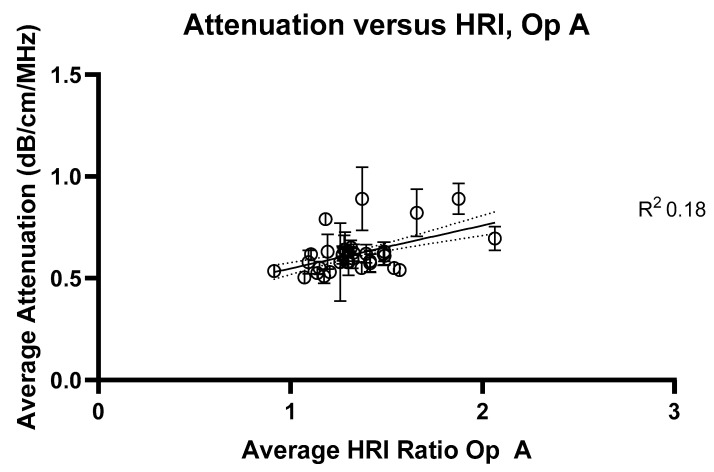
Average attenuation coefficients measured at 2 cm compared with hepatorenal index measurements for Operator A. R^2^ value = 0.18.

**Figure 7:**
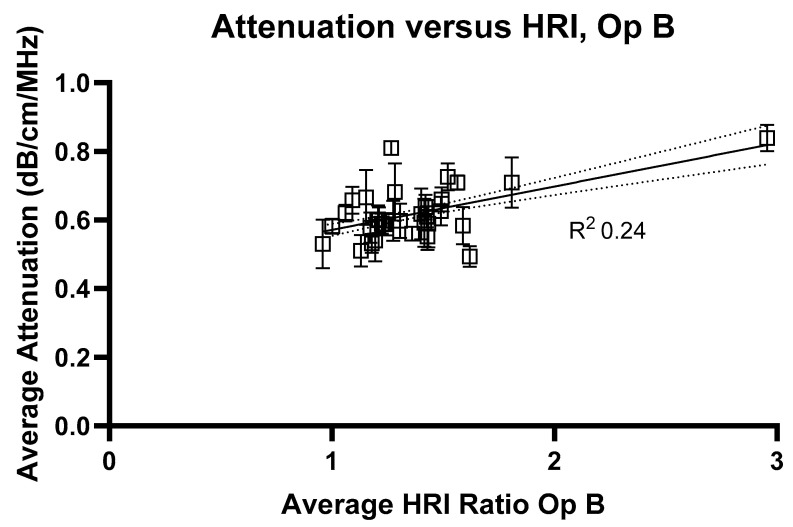
Average attenuation coefficients measured at 2 cm compared with hepatorenal index measurements for Operator B. R^2^ value = 0.24.

To fully investigate current methods of liver assessment, HRI measurements were also assessed for each volunteer. The comparisons of HRI to ATI measurements are presented in Figure 6 (Operator A) and Figure 7 (Operator B).

**Figure 8:**
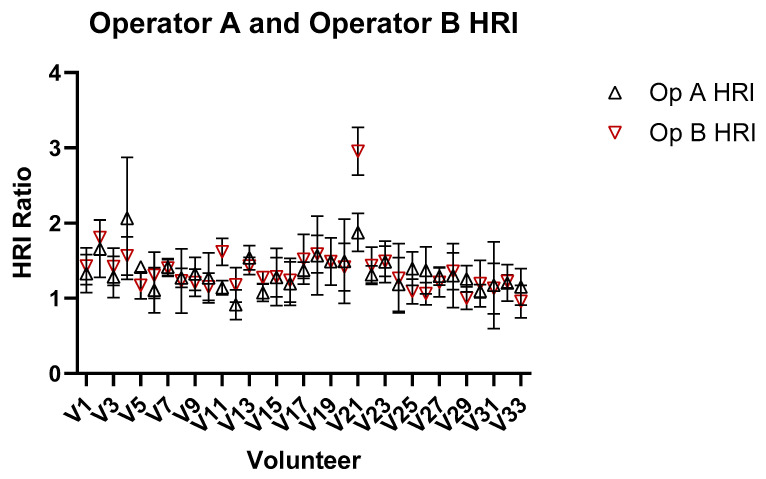
Comparison of HRI ratio obtained from images for Operators A and B of the liver and kidney.

**Figure 9:**
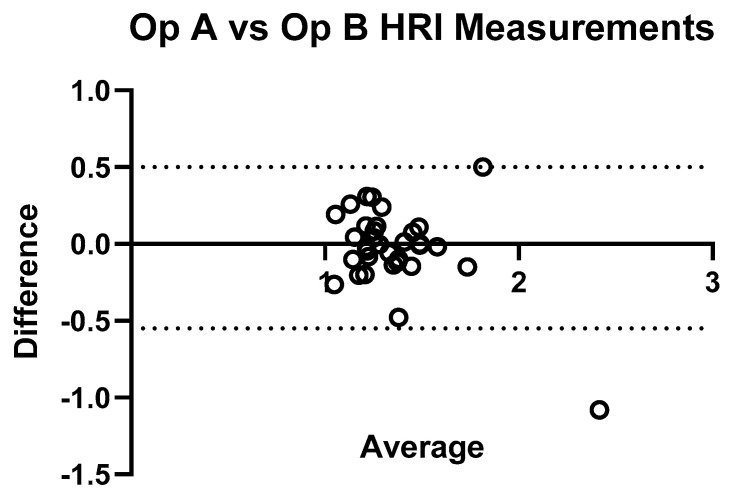
Bland–Altman plot illustrating inter-operator agreement for hepatic–renal index (HRI) measurements. The plot displays the difference between HRI values obtained by Operator A and Operator B (*Y*-axis) against their average (*X*-axis) for each subject (*n* = 33). The central solid line represents the mean difference, while the dashed lines indicate the 95% limits of agreement. Most points fall within these limits, indicating good agreement.

Figure 8 shows the comparison of HRI measurements for Operator A and B, and demonstrates significant inter-operator variability in measuring HRI values. Inter-operator agreement for HRI measurements was assessed using a two-way random-effects model ICC(2,1). The resulting ICC was 0.789 (95% CI: 0.622–0.887), indicating good reliability between the two operators. Figure 9 shows a Bland–Altman plot demonstrating the difference in measurements between Operator A and Operator B.

**Figure 10:**
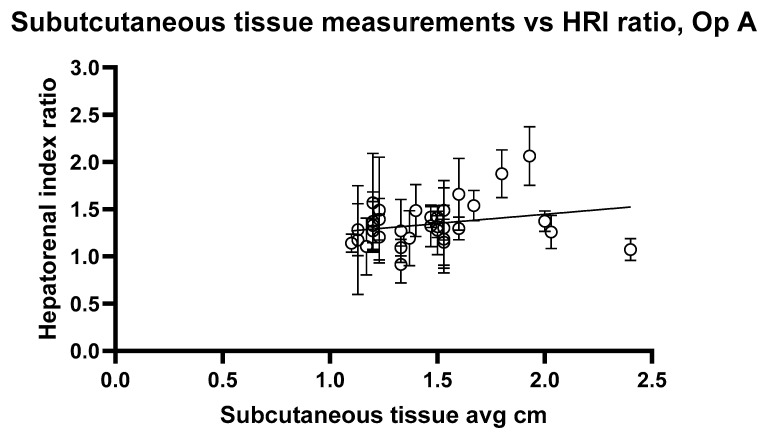
Hepatorenal index images for Operator A compared with the average subcutaneous tissue for each volunteer. Error bars represent the standard deviation for each volunteer. R^2^ = 0.02545.

**Figure 11:**
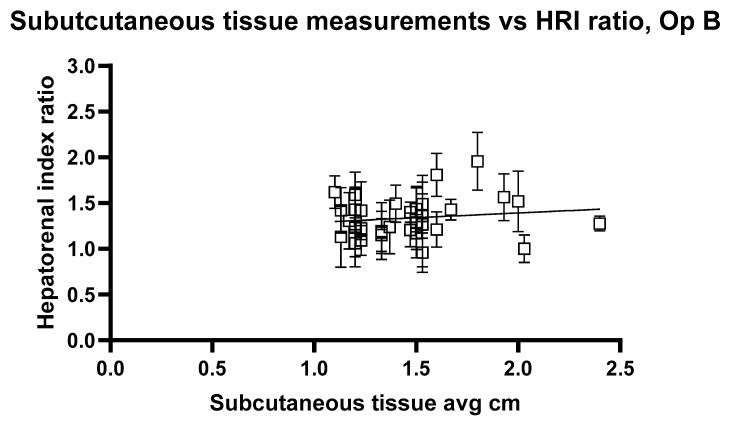
Hepatorenal index images for Operator B compared with the average subcutaneous tissue for each volunteer. Error bars represent the standard deviation for each volunteer. R^2^ = 0.02520.

Figure 10 and Figure 11 show the relationship between HRI values and volunteer subcutaneous tissue thickness and indicate that subcutaneous fat and HRI image values do not show a good correlation.

The analysis reveals a statistically significant positive relationship between subcutaneous fat and HRI values, as evidenced by the slopes of 0.1947 and 0.2199 for Op A and Op B images, respectively. Both slopes have *p* values < 0.05 (0.0407 and 0.0417), indicating that the observed relationships are unlikely due to random chance. Additionally, the 95% confidence intervals for the slopes (Op A: 0.008357 to 0.3811; Op B: 0.008386 to 0.4315) further confirm the significance of these relationships. However, the low R^2^ values (0.02545 for Op A and 0.02520 for Op B) indicate that subcutaneous fat accounts for only approximately 2.5% of the variability in HRI values, suggesting that other factors play a more substantial role in influencing HRI values. While the relationship exists and is statistically significant, it remains weak in predictive strength.

**Figure 12:**
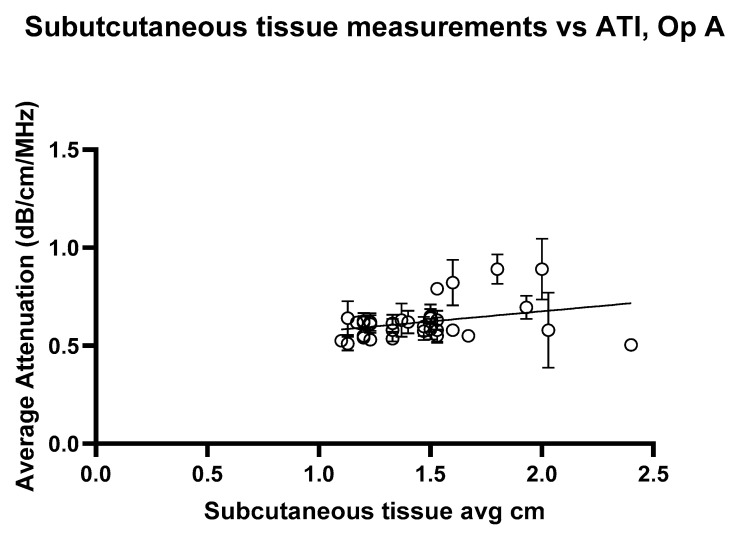
Attenuation images for Operator A compared with the average subcutaneous tissue for each volunteer. Error bars represent the standard deviation for each volunteer. R^2^ = 0.07276.

**Figure 13:**
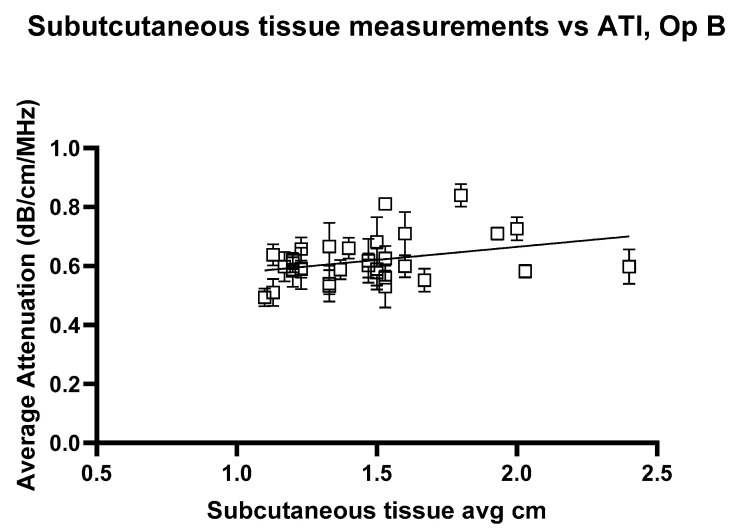
Attenuation images for Operator A compared with the average subcutaneous tissue for each volunteer. Error bars represent the standard deviation for each volunteer. R^2^ = 0.08939.

The linear regression analysis for ATI versus subcutaneous tissue thickness, Figure 12 and Figure 13, shows the regression slope was 0.1030 for Operator A, indicating a positive association between attenuation values and the measured variable. Despite this, R^2^ = 0.07276 indicates that only approximately 7.3% of the variability in attenuation values can be attributed to the subcutaneous thickness. For Operator B, the regression slope was slightly higher at 0.08858. Operator B’s analysis showed a slightly higher R^2^ of 0.08939, suggesting that the measured variable could explain approximately 8.9% of the variability in attenuation values. Both analyses highlight statistically significant yet weak predictive relationships, indicating that other factors likely contribute to the observed variability in attenuation measurements at shallow depths. These findings underscore the need for further exploration of additional variables to enhance the predictive power of attenuation imaging in clinical diagnostics.

## 4. Discussion

Ultrasound diagnostic imaging continues to play an increasingly pivotal role in the early detection and management of hepatic conditions. In the study presented here, attenuation imaging was conducted using a convex ultrasound probe, with measurements taken at varying depths in healthy volunteers. Depth significantly impacted attenuation coefficient values, with reductions observed as depth increased. This aligns with prior research indicating that ultrasound wave interactions with tissue lead to absorption and scattering, resulting in signal attenuation proportional to depth. Factors such as subcutaneous fat and region-of-interest (ROI) size further influenced the measurements, underscoring the importance of standardised protocols in obtaining reliable results. Previous studies have demonstrated that ATI values correlate strongly with liver steatosis grades (S0 to S3), reinforcing its capabilities as a diagnostic tool for characterising the severity of hepatic steatosis [11,17,18,22,23,24,25,26]. MASLD, encompassing a spectrum of conditions from simple steatosis to metabolic dysfunction-associated steatohepatitis (MASH) and cirrhosis, requires robust diagnostic protocols to facilitate appropriate intervention and monitoring. The findings presented in this pilot study underscore both the potential and limitations of ultrasound attenuation imaging as a diagnostic tool and highlight key factors that clinicians and researchers must address to optimise its use.

The data reveal a significant influence of depth on attenuation coefficient measurements, corroborating the work of Ferraioli et al. [16]. The attenuation coefficient values consistently decrease with increasing depth, a phenomenon attributed to the interaction between ultrasound waves and tissue properties. Specifically, wave absorption and scattering intensify as depth increases, resulting in greater energy dissipation and diminished signal strength returning to the transducer. Furthermore, reverberations arising from interactions between the ultrasound waves and multiple tissue boundaries compound this effect. This work, therefore, highlights that it is critical for clinicians to place the region of interest (ROI) at consistent depths in order to ensure accurate measurements, especially when performing longitudinal time-based follow-ups. The established manufacturer/WFUMB guidelines [16,27,28]. Initiating measurements at 2 cm below the liver capsule reflects these considerations; however, variability in patient anatomy, including differences in subcutaneous fat thickness, may render this guideline insufficient. Subcutaneous fat layers, varying from patient to patient, can lead to effective measurement depths ranging from 3 cm to 6 cm beneath the skin surface, further exacerbating variability in attenuation coefficients. This highlights the importance of tailored measurement protocols accounting for individual anatomical differences.

Larger ROIs encompass a broader tissue area and are prone to incorporating structures such as blood vessels, thereby altering the averaged attenuation measurements. Conversely, smaller ROIs offer more targeted assessments by isolating specific regions of liver tissue, but at the cost of increased variability due to reduced sampling size. These findings, consistent with those of previous studies [16,17], underscore the need for a balanced approach when determining ROI size. Optimising ROI dimensions to minimise artefacts while maintaining sufficient sampling remains critical for reliable and reproducible attenuation imaging.

The comparative analysis of ATI and HRI further emphasises the challenges inherent in current ultrasound-based diagnostic practices. Although HRI remains widely used in liver health assessments, the study revealed weak correlations between HRI and attenuation coefficient values, as evidenced by low R^2^ values across operators [10,11,12,23,29]. Given its susceptibility to variability and confounding factors, these results suggest that HRI may have limited utility as a standalone diagnostic tool for hepatic steatosis. In contrast, ATI offers depth-specific insights that may better capture the nuanced changes associated with hepatic steatosis. Despite being more robust than HRI, ATI still suffers from some variability. One possible explanation is variation in subcutaneous fat; however, in regression analysis, the low R^2^ value indicates that fat alone only explains a small amount of the variability. Thus, in addition to depth and fat, further investigation is required. Advances in automated software solutions, such as AI-driven ROI selection, could further reduce variability and improve consistency across practitioners.

Recent developments in AI-enhanced ultrasound imaging have demonstrated the feasibility of automated region of interest (ROI) determination across multiple clinical applications. Dicle (2023) highlights how AI, particularly convolutional neural networks (CNNs), can assist with image quality enhancement, lesion detection, segmentation, and classification in real time—offering a means to standardise operator-dependent examinations [30]. For example, in thyroid sonography, CNN-based systems demonstrated diagnostic performances equivalent to those of expert radiologists. Ko et al. reported AUCs ranging from 0.845 to 0.850 for CNNs versus 0.805–0.860 for radiologists [31], while Li et al. found higher specificity values for CNN models compared with human readers across multiple centre and large datasets [32]. Similar improvements in diagnostic consistency have been observed in breast ultrasonography [33] and liver lesion characterisation [34]. These findings suggest AI-driven tools hold promise in enhancing reproducibility and reducing reader-dependent discrepancies in ultrasound imaging workflows, such as those seen in hepatic–renal index (HRI) assessments.

Future research should explore integrating such technologies into clinical practice alongside developing refined guidelines that address the confounding effects of depth, ROI size, and subcutaneous fat layers.

From a technical perspective, the signal-to-noise ratio is a key determinant of measurement accuracy, particularly at greater depths. As ultrasound waves penetrate deeper into the tissue, the returning signal diminishes, making it increasingly challenging to distinguish the true signal from noise. This technical limitation compromises the reliability of attenuation coefficients obtained from deeper regions, particularly in heterogeneous organs such as the liver. Addressing this challenge requires advancements in ultrasound technology to enhance signal fidelity and improve the interpretability of attenuation data.

## 5. Conclusions

In summary, this pilot study reinforces the clinical relevance of ultrasound attenuation imaging whilst also examining limitations and areas for improvement. Depth-dependent attenuation, ROI size considerations, inter-operator variability, and the role of subcutaneous fat layers all warrant attention in the development of optimised imaging protocols. Further investigation into these factors, alongside exploration of nonlinear relationships and the integration of advanced imaging technologies, will contribute to the establishment of robust and reliable diagnostic practices for hepatic steatosis. Future studies require the addition of confirmed MASLD cases to fully explore many of the factors investigated in this paper, to understand the true ability of ATI and HRI evaluations. These insights serve as a foundation for future research and innovation, aiming to advance the efficacy of ultrasound-based liver diagnostics and improve patient care outcomes.

## Figures and Tables

**Table 1:** Volunteer characteristics, *n* = 33. BMI—Body Mass Index. The range of group characteristics is presented in brackets, with the average.

	*n* (Range)
**Age (years)**	34.52 (24.5–63.3)
**Sex**	19 (f)/14 (m)
**Weight (kg)**	67.3 (52–86)
**Height (m)**	1.72 (1.53–1.90)
**BMI (kg/m^2^)**	22.6 (18.0–28.0)
**Alcohol > 14 units/week**	3 yes/30 no

## Data Availability

The data of this study is available on request.

## Abbreviations

ATI	Ultrasound attenuation imaging
HRI	Hepatorenal Index
ROI	Region of Interest
AOI	Area of Interest
MRI	Magnet resonance imaging
MRI-PDFF	MRI proton density fat fraction
MASLD	Metabolic dysfunction-associated steatotic liver disease
NAFLD	Non-alcoholic fatty liver disease
